# Automating surveillance for healthcare-associated infections: Rationale and current realities (Part I/III)

**DOI:** 10.1017/ash.2022.312

**Published:** 2023-02-10

**Authors:** Erica S. Shenoy, Westyn Branch-Elliman

**Affiliations:** 1 Infection Control Unit, Massachusetts General Hospital, Boston, Massachusetts; 2 Division of Infectious Diseases, Department of Medicine, Massachusetts General Hospital, Boston, Massachusetts; 3 Harvard Medical School, Boston, Massachusetts; 4 Section of Infectious Diseases, Department of Medicine, Veterans’ Affairs (VA) Boston Healthcare System, Boston, Massachusetts; 5 VA Boston Center for Healthcare Organization and Implementation Research (CHOIR), Boston, Massachusetts

## Abstract

Infection surveillance is one of the cornerstones of infection prevention and control. Measurement of process metrics and clinical outcomes, such as detection of healthcare-associated infections (HAIs), can be used to support continuous quality improvement. HAI metrics are reported as part of the CMS Hospital-Acquired Conditions Program, and they influence facility reputation and financial outcomes.

The coronavirus disease 2019 (COVID-19) pandemic strained the resources of healthcare epidemiology and infection prevention and control programs, underscoring the need for robust systems-level support to optimize the ability of programs to respond to future system stressors. The rapid expansion of electronic health records (EHRs) presents an opportunity to implement automated measurement processes that theoretically reduce personnel resources required to complete healthcare-associated infection (HAI) surveillance requirements and that improve accuracy, reliability, and reproducibility. However substantial challenges and barriers to automating the process remain. In this review, we discuss current National Healthcare Safety Network (NHSN) definitions from the Centers for Disease Control and Prevention (CDC), and data quality requirements, EHR data structure, and leveraging structured data to support fully and partially automated surveillance processes. In two accompanying reviews, emerging technologies, cost and human resource considerations, and future directions are discussed.^1,2^

In 1963, Langmuir broadly defined infection surveillance as, “close observation to detect the early signs of infection … it implies maintaining a responsible alertness, making systematic observations and taking appropriate action when indicated.” When the term is applied to particular disease dates, surveillance means “the continued watchfulness over the distribution and trends of incidence through the systematic collection, consolidation and evaluation of morbidity and mortality reports… Intrinsic in the concept is the regular dissemination of the basic data and interpretations to all who have contributed and to all others who need to know.”^3^

In 2023, surveillance for HAIs contains many of the elements defined by Langmuir: systematic observation and data collection with reproducible and objective definitions, consolidation, and dissemination of the data with evaluation for temporal changes. Ideally, data collected through HAI surveillance processes can be used to inform practice and improve outcomes. Increasingly, these data collection processes are performed using automated data extraction methods. Surveillance is one of the pillars and core functions of infection prevention and control (IPC), facilitating the assessment of compliance with process measures, such as hand hygiene and adherence to standard and transmission-based precautions, as well as the assessment of outcome measures, such as HAIs.^4^

Many HAIs are preventable^5^ and costly.^6–8^ Increasingly, the performance of healthcare facilities on IPC metrics is tied to financial and reputational implications despite their unclear impact on clinical outcomes.^9–13^ Externally, HAI surveillance, measured most frequently as the standardized infection ratio (SIR), is used as a measure of hospital quality and for comparing facilities. Internally, HAI surveillance is a form of quality monitoring to detect problems and to deploy interventions in as close to “real time” as possible. Although improvements in HAI detection methods have decreased the time from event occurrence to detection, infection surveillance identifies outcomes that have already happened. Thus, infection surveillance is always retrospective, even if the surveillance occurs proximate to the occurrence of the infection or “in real time,” as it may be termed.

The ultimate objective of HAI surveillance is to collect and analyze critical data that can be used to improve clinical outcomes. For this conceptual benefit to be realized, several tenets must be satisfied. First, what is being measured must be important and actionable and should point toward potential improvement opportunities. Measuring events that do not negatively impact patients (or have potential downstream consequences) and measuring events that are not preventable with currently available tools will not translate into improvements in outcomes. Second, the events being measured must be well delineated, with objective and reproducible definitions that can be broadly applied. Third, definitions should reasonably capture what you want to measure. The ultimate aim of surveillance is to identify clinically significant infections. Given the inherent subjectivity in clinical diagnosis, there is always a tradeoff between capturing true infections and designing measurement processes that are objective, reproducible, and comparable across institutions. Surveillance definitions need to be clinically important and objective to be both clinically and operationally useful. Fourth, ideally, definitions are simple and easy to apply. As EHR systems continue to advance, it is tempting to move to HAI surveillance that is fully automated so that IPC efforts can be directed toward patient care activities rather than measurement.

As healthcare has transitioned to using EHR systems,^14^ opportunities have arisen to improve upon the accuracy and efficiency of HAI surveillance, to expand existing HAI surveillance to include a broader range of clinical conditions, and to expand the scope of clinical practices that benefit from surveillance activities, including responding to system stressors, such as outbreaks and pandemics. Full and partial automation of some components of HAI surveillance are integral to realizing these opportunities. However, despite much progress in transitioning surveillance to rely on structured EHR data, challenges remain. In this review, we describe the current state of automation in HAI surveillance with a focus on HAI surveillance defined by the CDC’s NHSN, as well as and the barriers to and benefits of full and partial automation.

## Definitions, standardization, and data quality

To ensure consistency and comparability across facilities and within a facility over time, HAIs, including infections with epidemiologically important pathogens acquired in healthcare settings, device-associated infections, and surgical-site infections (SSIs), are identified through the application of standardized surveillance definitions. Surveillance for HAIs requires accurate identification of the populations at risk for a particular HAI (the denominator), case ascertainment to identify infections that meet established criteria (the numerator), and in many cases, capture of a series of patient- and facility-level variables that are used for risk adjustment and other purposes to improve interfacility comparisons.

Surveillance definitions differ from clinically defined infections, and they are designed to be objective and reproducible within and between facilities. In the United States, the NHSN, used by greater than 16,000 healthcare facilities, has established requirements and definitions to guide HAI surveillance activities. The NHSN serves as the repository of HAI and other healthcare surveillance activities, and it is used both to comply with state and federal requirements for submission of HAI data and to track progress towards national goals.^15,16^

## Structural and technological challenges for fully automated infection surveillance

Within the EHR, data can be stored as structured data elements (SDEs) or unstructured data. SDEs are more amenable to automated surveillance strategies, and they include administrative data (eg, *International Classification of Diseases, Tenth Revision Clinical Modifications/Procedure Coding System*, or ICD-10-CM/PCS), microbiology data (eg, wound culture results), and pharmacy data (eg, antimicrobial use to identify treatment of infections). Clinical documentation, surgical and pathology reports, and imaging results are generally stored as unstructured data, which is less amenable to integration into currently available automated surveillance packages. However, emerging data-science strategies may be leveraged in the future to expand the spectrum of data available for real-time automated detection and reporting.^1,2^

Several SDEs are commonly used for measurement in various settings and as trigger tools for some surveillance activities to streamline the manual review process by reducing the total number of cases to review. Although in theory these SDEs could be used to create algorithms with a sufficient level of accuracy to “measure” infections without additional manual review, the reality of current systems differs. The ICD-10-CM/PCS coding system was designed to improve the accuracy of diagnostic codes, but unfortunately, the impact of this policy change has varied. For at least some conditions, the conversion from ICD-9 to ICD-10 worsened predictive value, and these administrative data are not sufficiently reliable on their own to be used for mandatory reporting and surveillance.^17^ Limitations have also been noted for how microbiological data are collected. Microbiology results can be unstructured and are frequently not standardized between institutions; thus, straightforward data extraction may not be possible.

Although SDEs are amenable to automated extraction, in many cases, they are insufficient for HAI reporting and identification, which effectively means that some component of manual review will be required for the vast majority of required HAI surveillance for the foreseeable future. Although the manual review process is time-consuming and requires substantial infection prevention resources and expertise, it also allows for more in-depth data collection and thus provides more information than simply the presence or absence of infection. The manual chart review process can also facilitate the identification of various factors that may have contributed to the occurrence of the HAI and thus can be used to determine how to improve infection prevention practices to prevent additional events from occurring and to guide local process improvement.

Despite challenges with currently available EHR structures and data, some HAIs, as defined by the NHSN, are inherently more suited to a relatively automated process, particularly those that are driven primarily by clearly defined laboratory results that are available as SDEs. However, even HAIs that are theoretically primarily defined using microbiology results, such as CLABSIs, may have specific exclusion criteria that necessitate manual adjudication of cases identified through automated processes.

## NHSN surveillance: Requirements and realities

One of the stated objectives of the NHSN definitions and reporting process is to utilize data that are readily available in EHRs to reduce the burden of surveillance for IPC, allowing resources that would otherwise be dedicated to surveillance to be applied to prevention of HAIs in other ways.^16^ Despite the development of standardized criteria that aim to use SDEs, the implementation of NHSN surveillance is resource intensive, even when using fully integrated commercially available surveillance systems. The process requires extensive validation, and frequent updates to the NHSN definitions mean that electronic algorithms require ongoing support from facility-supported information technology (IT) departments to ensure accurate reporting longitudinally. Although automated systems can theoretically alleviate some of the burden on IPC programs, validation and the need for ongoing updates may shift portions of the surveillance workload from IPC to IT without substantially reducing the overall personnel time required. However, although the impacts on personnel time are more limited than would be hoped, electronic systems can support improvements in accuracy of measurement, standardization of processes, and expansion of surveillance beyond traditional settings.

## HAI elements: Structured and unstructured data

The use of discrete data fields substantially improves the feasibility of automating surveillance processes. SDEs and SDE systems record patient and administrative data in discrete fields instead of narrative or free text. When patient identifiers, demographics, diagnoses, vital signs, and laboratory data are available as SDEs, automation is more likely to be successful because programming and informatics support needs are lower (Table 1). The use of natural language processing (NLP) to assess unstructured data can theoretically be used to assist in the automation of both numerator and denominator data. However, full realization of NLP-based surveillance strategies is limited by the complexity of unstructured data, including variable documentation practices, and the tendency for historical data to be copied forward in clinical notes, among other challenges.^18^

**Table 1.** Structured and Unstructured Elements of Denominator and Numerator Elements for NHSN Surveillance

NHSN Surveillance	Denominator and Risk Adjustment Variables (At-Risk Population Determination)		Numerator (Case Ascertainment)	
	Structured Data Element	Unstructured	Structured Data Elements	Unstructured
**Fully automated**				
LabID	Patient days	N/A	Time to microbiology result Microbiology result (CDI, MRSA)	N/A
**Mostly automated**				
VAE	Device (ventilator) days	N/A	Ventilator settings and changes over time (PEEP, FiO2) Antimicrobial agent and route of administration Microbiology results including application of definition of purulence, quantitative and semi-quantitative culture results (these may be unstructured)	Lung histopathology
**Partially automated**				
CAUTI	Device (indwelling urinary catheter) days	N/A	Vital signs (temperature) Microbiology results	Clinical symptoms (suprapubic tenderness, costovertebral angle pain or tenderness, urinary frequency, urinary urgency, dysuria)
CLABSI	Device (central-line) days	N/A	Vital signs criteria (temperature, hypotension) Microbiology results	Clinical symptoms (chills) Secondary infections Specific exclusion criteria (eg, documentation of patient directly injecting into the line, diagnosis of Munchausen Syndrome by Proxy)
SSI	ICD-10 PSC/CM codes Sex Age BMI Duration of operation	ASA score Wound class with or without diabetes	Microbiology results	Clinician diagnosis of infection Clinical signs and symptoms (eg, purulent drainage, dehiscence) Infection present at the time of surgery Imaging results

Note. NHSN, National Healthcare Safety Network; CAUTI, catheter-associated urinary tract infection; CLABSI, central line-associated blood stream infection; VAE, ventilator-associated event; PEEP, positive end expiratory pressure; FiO_2_, fraction of inspired oxygen; LabID, laboratory identified event reporting; SSI, surgical site infection; CDI, *Clostridioides difficile* infection; MRSA, methicillin-resistant *Staphylococcus aureus*; BMI, body mass index.

### Identifying denominators

Much of the attention to HAI reporting and measurement focuses on accurate identification of HAI cases. However, because the external use of HAI metrics is the basis for hospital comparisons and reimbursements, accurate measurement of the denominator (ie, the patient population at risk of an HAI) is also critical to ensuring accurate representation of a facility’s performance. As part of the CMS Hospital-Acquired Condition Reduction Program,^9^ facilities that are in the bottom quartile of performers across all metrics will have their reimbursements reduced by 1%. Hospital reputation is also positively affected by designations as “high performing” and negatively affected by “low performing” ratings. Additionally, reputational impact can translate into broader financial implications for the facility. Inaccurate denominator measurements have potentially steep consequences. If the denominator is inappropriately low, leading to a higher SIR, the facility could face financial and reputational penalties. Thus, substantial time and energy are often dedicated to ensuring accurate denominator ascertainment.

Most HAI denominators, such as urinary catheter days, central-line days, ventilator days, patient days, and operative volume, naturally lend themselves to automated surveillance due to the objective and often structured nature of the data fields that inform these variables. For HAIs that are based entirely on microbiology results, laboratory-identified (LabID) events, including *Clostridioides difficile* infection (CDI), and methicillin-resistant *Staphylococcus aureus* (MRSA) bloodstream infections, facilities must accurately identify total patient days and admissions to calculate an incidence per patient days to contextualize event rates. Surveillance for device-associated infections, including catheter-associated urinary tract infection (CAUTI), central-line–associated bloodstream infection (CLABSI), and ventilator-associated event (VAE), requires identification of the at-risk population, that is, patients with the device present for a minimum number of days.

*Device days.* For CAUTI, CLABSI, and VAE, the denominator is facility “device days,” defined as the total number of patient days for a given device. Under current guidance, these device days can be extracted and then submitted using a fully automated data extraction process. However, to submit electronically rather than manually generated denominator data to the NHSN database (ie, patient days, catheter days, central-line days, and ventilator days), facilities must undertake a time-intensive validation process that requires comparison of manually obtained data with data obtained via automated algorithms to ensure accuracy of reporting and that denominators are not under- or overcounted. Under current NHSN requirements, validation is often resource intensive because monthly automated denominator counts are required to fall within a 5% tolerance interval of the monthly manual denominator count (ie, generated by an individual conducting in-person observation of each patient and noting presence or absence of qualifying device) for a period of 3 consecutive months for each NHSN reporting location within the facility.^19^ Excursions outside the 5% interval require restarting the 3-month consecutive process. Once validated, electronically extracted denominator data are submitted monthly to the NHSN database as part of the facility’s monthly reporting plan, either entered manually or uploaded using the Clinical Documentation Architecture (CDA) import, .csv file import, or using the DIRECT CDA Automation protocol.

To date, we are not aware of any published studies that have quantified the costs of initial validation and ongoing data-quality checks as recommended by the NHSN, but at least 1 study reported variation in implementation of the validation requirements.^20^ Experience within our own institutions suggests that the validation process can be highly resource intensive. Furthermore, the benefits of the process are unclear, and some argue that the electronically measured device days are more accurate than the manually collected estimates and thus should be regarded as the “gold standard.” For example, Burke et al^21^ found that automated counts and manual counts of central-line days were discordant in 71% of the units in a 10-hospital system, and adjudication of discordant cases revealed that the automated count was correct 97% of the time. However, in another study, the manual collection process was more reliable.^22^ The high level of discordance in this hospital network also underscores the challenges of achieving sufficient agreement between the manual collection process and automated data extraction to satisfy NHSN requirements, and thus the need for ongoing IPC resources.

*Surgical volume and case mix.* The denominator for SSIs is the total number of eligible surgical procedures performed within a defined window. The identification of SSI denominators is completed using automated data extractions that rely on the ICD-10-CM/PCS and the current procedural terminology (CPT) coding systems to identify operative procedures that fall under the umbrella of NHSN surveillance activities. Maintaining current ICD-10 and CPT classifications is required, and the NHSN releases periodic updates to the list of procedures with mandated surveillance. Thus, ongoing informatics support is required for accurate denominator assessment.

Once procedures are identified, additional data to inform denominator data and risk adjustment must be included and are termed “denominator for procedure details.” These variables may be available as SDEs and thus are amenable to electronic data extraction methods if they are recorded in the EHR. These data include American Society of Anesthesiology (ASA) physical status score, NHSN-defined diabetes, duration of the operative procedure as defined by the Association of Anesthesia Clinical Directors (AACD), whether or not the procedure was emergency or urgent care, the use of general anesthesia, patient height and weight, documentation of inpatient or outpatient designation, closure (nonprimary or primary), use of a scope in the operative procedure using ICD-10-CM/PCS or CPT codes, whether the patient sustained traumatic injury prior to the start of the procedure, and wound class. The identification of automated sources for all these required variables and generating a file for submission to the NHSN requires facility-level expertise in informatics and IPC. Commercial software systems may have established programs to extract the necessary data elements from the EHR; however, local implementation requires significant subject-matter expertise, including approaches for managing missing data when elements are not recorded in the course of clinical care and for managing updates and changes to surveillance required by the NHSN.^23^ When all data elements are electronically available, similar to denominator data for device-associated infections and LabID events, surgical denominators can be extracted automatically from the EHR and uploaded to the NHSN database, either manually or as a file upload.

### Identifying numerators: HAI case ascertainment

HAI case ascertainment can be achieved through active manual review of all cases, through passive reporting of possible HAI cases to infection prevention departments for review, or through a trigger-based method in which a subset of cases is reviewed. Trigger-based surveillance can be based on general adverse-event detection systems, such as the Institute for Healthcare Improvement Global Triggers Tool^24^ or on specific event identification, such as microbiology results, antimicrobial orders, review of administrative data, or identification of key words in clinical notes or imaging reports. Each of these surveillance mechanisms may be supported by automated processes.^25^

HAIs that are most amenable to automation are those that include simple, objective criteria that can be translated into logic statements to support automated data extraction of SDEs. Such SDEs include those based on vital-sign data or simple laboratory definitions that do not require complex programming or manual review to interpret or classify results. HAIs that require additional information with subjective components (eg, clinician diagnosis of infection) or typically unstructured data elements (eg, imaging results, intraoperative findings, or in some cases microbiology results) are less amenable to a fully electronic surveillance program. However, semiautomated processes can be employed to streamline the chart review and manual confirmation of infections.

Although fully automated surveillance may be possible for straightforward measurements, such as LabID events, measurement of other HAIs requires manual review, typically completed by an infection preventionist. The need for manual chart review has several factors. First, surveillance definitions are designed to be objective, but many HAI definitions include some element of subjectivity. For example, the diagnosis of superficial SSIs includes “clinician diagnosis of infection” as one of the criteria.^26^ Different clinicians often disagree about whether an infection is present, and a computer algorithm cannot decipher potentially conflicting clinical documentation. Some elements of surveillance definitions, while technically possible to store in a structured format for later extraction, are not stored as such due to variation in the extent of EHR integration. For example, the use of free-text reporting for unstructured microbiological results makes reliable electronic surveillance nearly impossible. Leveraging SDEs using various trigger tools (eg, the presence of a microbiological sample or documented order) can be helpful for streamlining cases for review. However, this process will only reduce (not eliminate) the need for manual review because microbiology results, not orders, are the data needed to ascertain whether an event occurred.

## Fully automatable: LabID events

The notable exceptions to the need for manual adjudication of HAI cases are the LabID events, which are defined as a positive microbiology result within a certain window of time after admission (eg, blood cultures for MRSA, and a positive assay for CDI). Assuming that microbiology results are reported using SDEs (eg, MRSA codes based on organism and susceptibility or other SDE and not entered into the EHR as free-text data), these structured fields along with admission, discharge, and transfer (ie, A/D/T) fields, are entirely extractable from EHR systems. Thus, surveillance for these events can, with the appropriate and validated electronic surveillance system in place, be conducted without any manual review.

## Mostly automatable: VAE

VAE surveillance is one example of NHSN surveillance that includes almost entirely SDEs in the EHR. Thus, it is, theoretically, almost fully automatable. These SDEs include ventilator settings (and changes in ventilator settings over time), temperature, white blood cell count, administration of specific antimicrobials, and microbiological diagnoses (including detailed specimen types, and quantitative measurements). Surveillance systems that can extract these data elements and apply if–then logic can approach full automation. The complexity of if–then statements makes this definition ideally suited to automated surveillance, which can also avoid the errors noted when surveillance is applied manually.^27^

Full automation for VAE is stymied by caveats to the requirements for possible ventilator-associated pneumonia (PVAP). PVAP requires that 1 of 3 criteria be met, and criterion 3 includes lung histopathology, which is generally not recorded in a structured manner amenable to straightforward automated data-extraction methods. Institutions with advanced informatics support may be able to apply NLP or machine learning to evaluate and classify lung histopathology findings, which would allow full automation, but substantial upfront and ongoing informatics support would be needed to ensure ongoing accuracy of the automated measurement tools over time.

A hybrid approach to streamlining the process could include automated data extraction of the relevant histopathology results paired with the SDEs to streamline but not eliminate the need for manual review of this element of the definition. Nevertheless, if the case meets any of the other 2 criteria, or 1 of 3 other possible tests as part of criterion 3, the case could be designated as a PVAP without any chart review required. Thus, in practice, the manual review required to support PVAP reporting is generally quite limited, and the benefits and informatics resources required to approach full automation must be weighed against the additional complexity of achieving a completely automated surveillance system.

## Partially automatable: CAUTI, CLABSI, and SSI

For other device-associated infections and SSI surveillance, although partially automated extraction of EHR elements that define an infection are possible, chart review by an individual trained in NHSN surveillance is necessary, and surveillance for these HAIs should be considered partially automatable.

### CAUTI

The NHSN definition of CAUTI requires that 3 elements be met: (1) the presence of an indwelling urinary catheter for at least 2 days in an inpatient location on the day of the event; (2) the presence of at least 1 of 6 clinical signs or symptoms (fevers, suprapubic tenderness, costovertebral angle pain or tenderness, urgency, frequency, or dysuria); and (3) a urine culture meeting specific organism and quantity criteria.

Automated surveillance systems can usually accurately assess whether the patient meets elements 1 and 3; however, depending on the way urine cultures are reported in a laboratory information system, automated assessment for element 3 may be challenging. With the exception of temperature data (typically recorded as structured vital sign data), ascertainment of clinical symptoms, which are generally only captured in clinical notes as unstructured data, are difficult to extract reliably using existing automated technology. Although it is theoretically possible to use simple text-note searches and NLP to identify key words and phrases such as “urinary frequency,” documentation practices vary. Also, negative findings (eg, “patient denies urinary frequency,” “no urinary frequency, “-urinary frequency,” “history of frequency”) are often included in clinical notes and are often copied forward, which complicates the operational accuracy of this theoretically attractive approach.^18^ Thus, unless a patient meets the criterion of fever, chart review or advanced data extraction methods are necessary for all patients who meet elements 1 and 3. This additional review must assess for reports of suprapubic tenderness, costovertebral angle pain or tenderness, urinary urgency, urinary frequency, or dysuria. Multiple examples of automation of CAUTI surveillance have been reported.^28–30^

### CLABSI

The NHSN surveillance definition for CLABSI requires that 3 elements be met: (1) presence of a central line for at least 2 days following the first access of the central line in an inpatient location and during the current admission; (2) recovery of a recognized bacterial or fungal pathogen from a blood culture; and (3) the organism identified in the blood is not related to an infection at another site.^31,32^

Automated surveillance systems can usually accurately assess whether the patient meets elements 1 and 2; however, the exclusion of infection at another site as well as assessment for other exclusion criteria is challenging, and in some cases, impossible. For example, a patient meeting all criteria for a CLABSI but with documented evidence of central line access by a nonclinical provider is excluded. This type of complexity is not directly ascertainable from the EHR without substantial manual review. Despite these barriers, several examples of partial automation have been reported.^33–35^

### SSI

Case ascertainment of SSIs can proceed either through chart review of all eligible procedures or through chart review of a subset of procedures often termed “trigger review” in which the trigger-prompting review can include readmissions or repeated procedures during the at-risk period, presence of laboratory, imaging, and/or other diagnostic tests. The NHSN provides examples of active, patient-based, prospective surveillance required, including concurrent and postdischarge methods. SSI case ascertainment is labor intensive for infection preventionists, who must apply the NHSN criteria to establish the following elements: level of infection (ie, superficial, deep, or organ space), whether primary or secondary, the specific criteria met for SSI (from a list of signs and symptoms, laboratory diagnostics, and clinical diagnoses, some of which are contingent upon the specific site of infection), report specific pathogens (if identified) and antimicrobial resistance profile, and whether the SSI contributed to death. Despite detailed instructions provided by the NHSN, concerns regarding subjective application of definitions have been raised and discrepancies have been noted when comparing NHSN outcomes to other surveillance initiatives, notably the American College of Surgeons National Surgical Quality Improvement Program (ACS NSQIP).^15,36,37^

## Benefits of automation: Speed, accuracy, and efficiency

Despite the requirement for chart review to apply the NHSN definition for device-associated infections and SSIs, automating aspects of surveillance can, through extraction of the available electronic elements, restrict the possible HAI cases that require chart review to only those that would otherwise meet the NHSN definition. In the CAUTI example, by applying elements 1 and 3, an automated system limits the total cases that require chart review. Automated data extraction systems also reduce the opportunities for human error in applying the definition. In the case of CAUTIs, they prevent errors in calculating duration of the indwelling urinary catheter in relation to the urine collection date and errors in interpreting urine culture results because they are assessed through the automated application of the surveillance definition. For VAEs, the complex calculations related to changes in ventilator settings in relation to the timing of other elements of the definition are prone to human error, which is mitigated entirely through automated calculation. Automated surveillance systems that organize chart review in a way that presents the relevant data in a single location for the infection preventionist conducting the chart review can increase the efficiency of surveillance. For example, automated SSI surveillance systems that organize operative notes, microbiological data, recent admissions, and other data in a single location for chart review can streamline case ascertainment. Additionally, automated surveillance programs that aggregate the case characteristics in the required format for upload into the NHSN database reduce the overall burden of surveillance.

In these examples, references to automated surveillance systems that allow for extraction, organization, and presentation of possible HAIs for review, as well as generation of case files for submission to NHSN, include both commercial and in-house systems. As the penetration and sophistication of commercial EHRs has grown, so too have embedded surveillance systems that aim to increase the accuracy and efficiency of NHSN and non-NHSN surveillance. For example, Epic software (Epic, Verona, WI) includes an infection control module (ie, “Bugsy”) and a module focused on inpatient admissions, discharges, and transfers (ie, “Prelude”). None of these commercially available systems, however, are “plug and play” systems. They require local integration and validation, which is time-consuming and costly. One advantage of commercial systems is the application of structured updates in response to changes from the NHSN, which are generally included in the support package. Again, these updates require local integration and validation and maintenance of facility-level IT resources working closely with IPC teams (Table 2).^38^

**Table 2.** Benefits, Downsides, and Considerations for Automated Infection Surveillance

Benefits of Automated Surveillance	Downsides of Automated Surveillance	Additional Considerations
**Time and resources**		
Potential to save infection control practitioner time once stably implemented	Increases need for IT support, which is challenging, particularly for smaller facilities	Costs of future saved IP time need to be weighed against increased IT costs
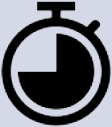	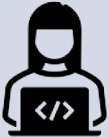	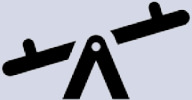
Frees up IP time for other activities, such as education, bedside rounds, pandemic responses	Initial validation requires substantial upfront IP resources. Updates and changes to HAI definitions require ongoing resources	During implementation, consider trade-offs between complexity of electronic tools and time-saved. Collaboration with IT is essential
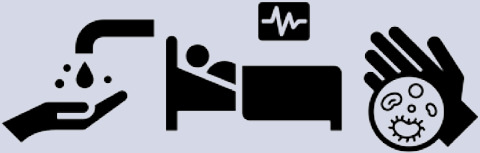	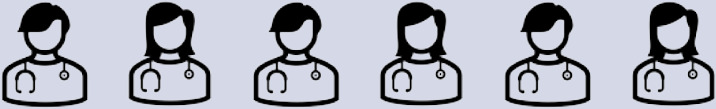	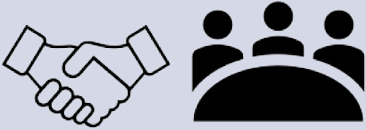
**Reliability and reproducibility**		
Objective measurement, potentially improving intra- and interfacility comparisons	Most HAI definitions require at least some manual review, introducing subjectivity and reduces intra- and interfacility comparisons	Reliability and reproducibility are challenging for some HAI (eg, SSI) due to variation in surveillance processes
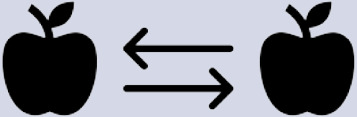	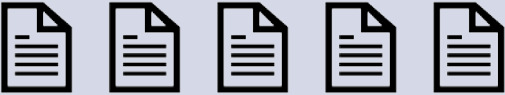	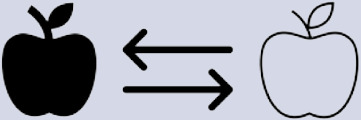
Most studies suggest automated infection surveillance estimates are more accurate than manually derived estimates	Some HAI elements have limited automation potential due to how the EHR data are organized	Natural language processing and machine learning may facilitate automation, however, requires complex and ongoing IT support
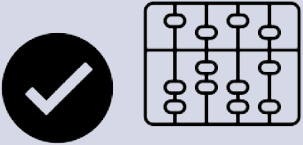	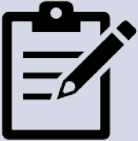	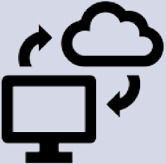
**Real-time quality improvement and evidence generation**		
Automated surveillance enhances our potential to link datasets (eg, clinical and genomics data, antimicrobial resistance profiles and treatment) to improve detection and response	Most automated infection surveillance activities occur within a facility or healthcare network. Without access to truly longitudinal data, detection strategies will always be limited	Automating surveillance provides the opportunity to achieve a “learning healthcare system” for real-time quality improvement
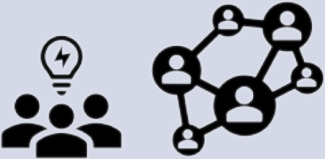	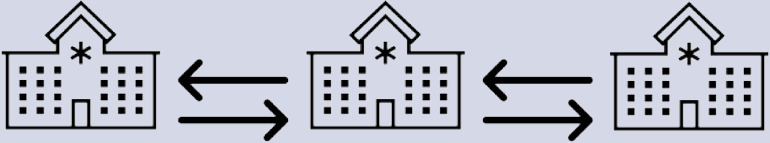	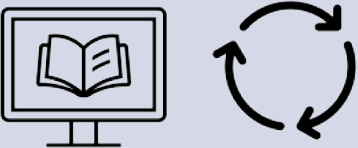

Note. EHR, electronic health record; HAI, healthcare-associated infection; IP, infection preventionist; IT, information technology; SSI, surgical-site infection.

In pursuit of these goals—and fully automated surveillance—it is important to consider unintended consequences and incentivizing behaviors that lead to events not being “counted.” For example, SSI surveillance definitions currently include a variety of data elements, including clinician diagnosis and imaging findings, that limit the potential for full automation. Switching to a simplified definition that includes only microbiology results would increase the potential for fully automated processes. However, doing so would violate several tenets of HAI surveillance and could potentially lead to unintended negative consequences for patient care. Using only microbiology results misses many clinically important cases, and prior research has suggested that the sensitivity of microbiology results–driven surveillance ranges from 33% to 71% sensitivity.^39^ This wide sensitivity range raises questions about standardization and reproducibility across institutions. Using a definition that only uses microbiology results to define the outcome may have substantial negative impacts on patient care. If the simplified definition leads to fewer microbiology cultures being collected for the purposes of avoiding being “counted,” then patients may receive worse care due to empiric, rather than culture-driven, antimicrobial therapy. Thus, goal of surveillance—to identify problems that lead to improvements in clinical care—would not be fulfilled.

## Future directions

As shown during the COVID-19 pandemic, IPC is pivotal to maintaining the safety of healthcare is often underresourced.^40,41^ The IPC workforce is facing daunting challenges of ensuring a pipeline of trained healthcare epidemiologists, infection preventionists, and support staff. They must simultaneously respond to increasing demands for expanded surveillance both in acute-care settings as well as across the continuum of care, including ambulatory, rehabilitation, long-term care, and home-based care. Meanwhile, an increasing proportion of healthcare has moved to these non–acute-care settings where IPC resources have lagged.^42^ Current payment and reimbursement staffing models do not have any “slack” built into the model; thus, even departments that are relatively well supported during nonemergency times do not have the capacity to absorb a higher workload when emergencies occur. New staffing models and strategies for reducing current responsibilities are needed to maintain healthcare quality, regardless of external and unpredictable pressures. To the extent that aspects of surveillance can be fully or partially automated, gains in efficiency and timeliness have the potential to support IPC and alleviate the current workload, expand the breadth of surveillance, and focus efforts on quality improvement and infection prevention initiatives. Realization of these gains, however, requires investments in validation and integration as well as continuous maintenance of electronic surveillance systems. Frequent reassessment of surveillance definitions at a policy level will be needed to ensure that they are capturing appropriate outcomes.

IPC subject-matter expertise is limited and should be focused on the highest-level tasks that cannot be automated. Investments in technology, training, and teams to move surveillance and prediction forward and harness the potential of the EHR to maximize patient safety are also needed. Although NHSN surveillance is the cornerstone of HAI surveillance, automated surveillance has a role far beyond the detection of HAIs. Using the EHR has great potential to develop and validate candidate surveillance definitions, to integrate NLP into surveillance, and to use the EHR to predict risk of HAI to target interventions. Although we cannot predict a timeline for achieving this potential, the future of automated infection surveillance and the promise it holds are discussed separately in two companion reviews.^1,2^
